# The Health Impacts and Life Challenges Caused by the COVID-19 Pandemic on Hong Kong Chinese Women

**DOI:** 10.3390/ijerph19095115

**Published:** 2022-04-22

**Authors:** Maria Shuk Yu Hung, Liliane Chui King Chan, Sisi Pui Shan Liu

**Affiliations:** 1 School of Nursing, Tung Wah College, Hong Kong, China; 2 Hong Kong Federation of Women’s Centres, Hong Kong, China; liliane.cck@gmail.com (L.C.K.C.); sisi.liu@womencentre.org.hk (S.P.S.L.)

**Keywords:** COVID-19 pandemic, health impacts, life challenges, Hong Kong, women, qualitative

## Abstract

The COVID-19 pandemic has caused a massive global crisis. The adverse impacts on Asian women, including Hong Kong Chinese women, have been considerable. The pressure on Hong Kong women is immense due to cultural, social, familial, and personal responsibilities. This study aims to illustrate the health impacts and life challenges for Hong Kong Chinese Women during the pandemic. An interpretive phenomenological approach with purposive sampling was adopted. Semi-structured, face-to-face, in-depth interviews were conducted from August 2020 to January 2021. Twenty-five women participated in the interviews, lasting an average of 48 min. The transcribed interviews were analyzed using interpretative phenomenological analysis. The core theme identified was “Perceived family caregiving as paramount self-obligation in times of the pandemic”, in the context of the role of daughter, wife, or mother (or a combination). Three interconnected themes have been identified in individual, relational, and external contexts: deterioration of personal health, unfavorable to family relationships, and adaptation to social challenges. Eight subthemes have emerged related to health impacts and life challenges. The pandemic has increased women’s perception of their caregiver roles in the family, but it has diminished their quality of life. The promotion of strategies and activities that could enhance women’s physical, psychological, emotional and social quality of life is recommended.

## 1. Introduction

The COVID-19 pandemic has been causing global human health, social, economic, and environmental crises since late 2019. Up to mid-April 2022, about 500 million and more than 6 million people had been infected and died, respectively [1]. The United Nations [2] has highlighted the impact of COVID-19 on women, including the complex negative economic consequences, adversely affected health, demands for increased unpaid care work, etc. These adverse impacts on women, and especially on Asian women, have been substantial, as these women have traditional and cultural responsibilities as families’ primary informal caregivers. Previous evidence has shown that women in China with parental caregiving obligations had poorer self-reported health than those who were not caregivers [3]. During COVID-19, the closing of schools to limit disease transmission has placed extra care demands and workloads on women, who are forced to care for their children at home. The increases in care demand have probably raised stress levels. This could affect women’s mental health and employment opportunities [4]. The significant reductions in financial status due to the pandemic have further induced worries and life quality changes [5,6]. A recent review identified the considerable risk of specific adverse health outcomes negatively affecting women’s health during the COVID-19 pandemic [7].

Although Hong Kong citizens have experienced various previous public health emergencies [8,9], e.g., the SARS and H1N1 pandemics, evidence has shown that Hong Kong citizens’ emotional, social, and economic status has been unavoidably affected by COVID-19, especially women [10,11]. A local survey assessed 126 middle-aged women who had experienced a psychological burden during SARS in 2003; their emotional distress was primarily related to financial losses and risk perception [8]. Most respondents (77%) were afraid of contracting the disease, and about two-thirds (70%) limited their social activities more than usual. A quarter of respondents reported restless sleep. Forty percent worried about their financial situation and their reduced household incomes [8]. Another local survey found that uninfected citizens with moderate anxiety levels and higher risk perceptions were more willing to endure thorough precautionary measures to prevent SARS infection [9]. Furthermore, those who had severe anxiety and feelings of helplessness may take inappropriate and potentially harmful remedies. In early 2020, the COVID-19 pandemic triggered panic in communities, leading to the purchasing of personal protective and disinfectant items [12]. The authors of this paper also conducted an initial online survey of 417 women aged 18 years or above in Hong Kong, seeking to assess the impacts of COVID-19 on this group [10]. The results show a high percentage of negative emotions such as stress (32.2%), anxiety (42.4%), and depression (44.9%), with significant negative correlations between emotional state and different aspects of quality of life [10]. Concerning the evidence that public health emergencies affect women most severely, this in-depth qualitative study aimed to understand the health impacts and life challenges related to COVID-19 on Chinese women in Hong Kong. The objectives were to explore their experiences of psychological and physical health impacts, and to identify the life challenges associated with caring for family during lockdown. The findings provide useful information that could assist local government officials, non-government organizations, and healthcare professionals in designing appropriate support systems and activities that will assist women during or after a pandemic.

## 2. Materials and Methods

### 2.1. Design and Participants

This study adopted a qualitative phenomenological approach. The ultimate goal of this method is to illustrate, comprehend and explain the informants’ experience [11]. The purposive sampling of subjects offering high amounts of information was employed in this study. Each participant could provide their experiences and share their viewpoints [13]. The subjects invited were the 417 women who had participated in the previous online survey [10], with the following inclusion criteria: Chinese females aged over 18, living in Hong Kong, who could understand Cantonese. Those who voluntarily agreed to participate in this extended study were called on the phone to invite them to participate in the individual interview.

### 2.2. Ethical Considerations

Ethical approval was obtained from the Research Ethics Sub-Committee of the researcher’s institution (Ref. No. REC2020071). The study was implemented based on the general principles of the Declaration of Helsinki. The detailed information of the structure and purpose of the study was given to the potential participants. Before the interviews, written informed consent was received from each subject. They had the right to refuse to join, and could refuse to answer any questions during the interviews. All the research information was kept confidential, and the anonymity of the participants was guaranteed.

### 2.3. Data Collection

After a preliminary quantitative analysis of the above-mentioned online survey [10], the participants’ emotional state, life experiences, and self-perceived coping ability and strategies were queried via guiding questions. Semi-structured, face-to-face, in-depth interviews were commenced in late August 2020. Semi-structured interviews are the most appropriate data collection method for interpretative phenomenological analyses [13]. Through semi-structured interviews, researchers were able to modify the initial questions based on participants’ responses, and explore any meaningful and significant content that emerged [13].

The interviews were conducted at the centers of different districts in Hong Kong that were familiar to the participants. The rooms used were comfortable, quiet, and free from distractions. Following the Hong Kong government’s policy of social distancing, only one-to-one interviews with appropriate distancing and adequate disinfection measures were performed. Before the interview, casual conversation was undertaken with the participants, and general questions were asked to learn more about them and establish a level of trust. For those women who brought their children, the center staff offered assistance in taking care of them in another room. This allowed both the participants and the interviewer to engage in a confidential and natural conversation without interruption. The interview’s purpose, how the information would be used, and the estimated duration of the interview were also explained. Consent for audio recording was also sought.

An interview guide was developed to aid in our understanding of the participants’ life experiences during the COVID-19 pandemic. For instance, “How did the COVID-19 pandemic affect your health and daily life? Please describe the unforgettable feelings or experiences in the past few months that COVID-19 caused.” Participants were asked to relate their feelings and experiences related to the health impacts and life challenges of COVID-19. They were encouraged to freely express their views and concerns. The interviewers were careful when asking questions and expressing responses, maintaining a friendly atmosphere and good rapport in order to encourage the participants to share their personal experiences. The researcher remained aware of her own preconceptions and personal experiences regarding COVID-19, and did not communicate these to participants, as this may have influenced their views. Subsequent probing and clarification questions were asked if required. Any coping strategies the participants used during the pandemic were also asked about.

The participants’ emotional conditions were closely observed throughout the interviews. Five of them experienced emotional distress, and three began to cry. Appropriate supportive actions, e.g., a short period of silence or rest, were undertaken. If required and the participant consented, they were referred for further support, such as psychological and financial counseling or legal advice on marital issues. The interviews lasted from 30 to 90 min, with an average of 48 min. The interviews yielded a valuable in-depth understanding of participants’ experiences during the pandemic. All the audio-recorded interviews were then transcribed for analysis. Field notes were taken to document the participants’ demographics, emotions, gestures and physical expressions, and the researcher’s impressions during and after the interviews. These notes were later incorporated into the verbatim transcripts. The researchers reviewed the first semi-structured interview transcript for interview skills, text accuracy, and overall understanding, and made minor adjustments before the following interviews.

### 2.4. Data Analysis

The data analysis was based on interpretative phenomenological analysis (IPA) [13]. The purpose of IPA is to recognize the essential subjective meaning of a participant’s experience of an event, instead of quantifying the data. The researchers sought to understand and interpret the participants’ personal, psychological and social world through IPA [13]. A step-by-step process was utilized to explore the participants’ perceptions [13]. The researchers explored themes derived from the transcription of the first participant’s interview, linking the themes and then progressing with the analysis of other participants [13]. Two team members individually read through the transcribed interviews and notes, and they wrote comments on the transcript to help identify recurrent ideas and expressions. Meaningful sections were further organized and categorized according to their characteristics. The researchers then sought themes and subthemes that would reveal the similarities and natural variations among individual participants, forming relationships within the data. The analysis was then continued with other participants. The researchers’ pre-existent understanding and knowledge regarding the pandemic assisted them in forming interpretations during the data analysis. After 24 participants had been interviewed, data saturation occurred. The researchers continued to interpret and refine the main themes and subthemes. Then, one more participant was recruited for an interview in January 2021 to verify these themes and subthemes.

## 3. Results

Of the 25 women participants, most (72%) were aged between 30 and 39 (40%) or 60 and 69 (32%). Fifteen were married, and six were separated/divorced. In total, 21 (84%) participants had caregiving responsibilities with children and/or elderly parents, and 8 (32%) had to care for two family members. Eight (32%) were housewives, and five (20%) were retired. The demographic information of these participants is summarized in Table 1.

The impact of COVID-19 on the Hong Kong women’s health and lifestyles is illustrated through the core theme, the three other themes, and the eight subthemes shown in Table 2. The core theme was “Perceived family caregiving as paramount self-obligation in times of the COVID-19 pandemic” as a daughter, wife and/or mother (or in combined roles). Three other interconnected themes arose in individual, relational, and external contexts, respectively: deterioration of personal health, unfavorable to family relationships, and adaptation to social challenges.

### 3.1. Deterioration of Personal Health

The first theme, “deterioration of personal health”, relates to the individual impacts on personal health. The two subthemes included emotional and psychological distress due to fear of COVID-19’s consequences, and physical exhaustion in performing the family caregiver role during COVID-19.

#### 3.1.1. Emotional and Psychological Distress Due to Fear of COVID-19’s Consequences

In relation to this subtheme, almost all participants (22 out of 25) expressed emotional and psychological distress due to the fear of themselves or family members becoming infected, e.g., unhappiness, worries, anxiety, stress, and depression. Apart from the fear of the direct consequences of being infected, the participants also expressed worries and concerns about the indirect impacts on their health and their family, e.g., financial losses. A mother with two children who was separated from her husband verbalized her fear of being infected, and its consequences.

*I’ve been under tremendous stress and anxiety. I’m a private tutor of school children and should have frequent contact with them and their parents. I was afraid that I would get infected or my kids would be infected as they followed me to my workplace. If I got infected, I could not work, and my income would be affected.*

(P17)

A retired participant expressed that her fear of contracting coronavirus led her to experience physiological symptoms that affected her physical wellbeing. She commented as below:

*I had gone to the places with confirmed cases. I’ve inadequate masks. I’m afraid I got infected and then transmitted it to others. I was so scared that I would die. I started to have a stomachache weeks ago and could not sleep well.*

(P1)

#### 3.1.2. Physical Exhaustion in Performing the Family Caregiver Role during COVID-19

Among the 25 participants, 21 were caregivers of family members, and 8 had responsibilities of care for two family members. The participants mentioned additional caregiving responsibilities that had arisen under the lockdown policy put in place during the pandemic, further aggravating their burdens. Seventeen participants experienced physiological symptoms of palpitations, inadequate rest, poor sleep quality, etc., primarily due to their additional family caregiving roles.

A housewife with two small children shared her daily experience as follows:

*I have a boy, aged five, and a girl, aged one. The kindergartens have been closed in the past few months due to the social distancing and lockdown policy. My son used to learn at the kindergarten during the daytime on weekdays. Apart from my busy family routines such as meal cooking, clothes washing, and extra household cleaning …, I should take care of them also. They are active, energetic, and playful at home. You know, just like having a battle every day! I’m so tired and exhausted every day. I cannot sleep well and have sufficient rest.*

(P16)

A few participants experienced severe physical impacts, including weight loss, chest pain and exhaustion, when performing their intense or continuous family caregiving roles. A retired lady who lived with and cared for her husband and daughter verbalized her recent challenges as follows:

*My husband is old and has a mobility problem, and my daughter is developmentally disabled. Due to the pandemic, the elderly daycare center and the shelter workshop were closed. They could not go there on weekdays and must stay at home. I take care of them by myself all around the clock without a break. I’m so exhausted. It’s too difficult for me. I have had insomnia with a 3 kg weight loss recently.*

(P8)

Although this lady voiced her difficulties, she stated she was still willing to care for her loved ones until such time as she was unable.

### 3.2. Unfavorable to Family Relationships

The second theme, “unfavorable to family relationships”, regards the impacts on the relationship with the family as a daughter, wife, or mother. The three subthemes identified were regrets of unfulfilled filial responsibilities, unhealthy marital relationships, and tension in parent–child relationships due to the prolonged pandemic. All these subthemes strongly reflect the traditional family values of Chinese women.

#### 3.2.1. Regrets of Unfulfilled Filial Responsibilities

Due to social distancing measures and the lockdown policy, elderly care centers were also closed. Six of the twenty-five participants stated they could not visit or take care of their sick family members, especially their parents who did not live with them in Hong Kong or mainland China. The participants’ comments reveal that filial piety and parental care are significant traditional concepts deeply rooted in their minds. For instance, they exemplify the Chinese values of caring for parents and being present as a family during parents’ final moments.

One participant’s experiences speak of a feeling of regret about unfulfilled filial responsibilities, as follows:

*My father-in-law fell from a height with a broken leg a few months ago in China. He was admitted to the hospital for an operation. We (participant and husband) should have, but we could not go there to care for him due to the lockdown. Our children and we missed him so much!*

(P16)

Another participant expressed the painful experience, and the guilty feelings associated with it, of not being present in the hospital during her father’s last moments due to the lockdown policy.

*I was so guilty about my father’s death. In June, my father’s health deteriorated suddenly. He was admitted to a hospital in mainland China. Due to the lockdown policy, we could not take care of him or bring him back to Hong Kong for treatment. We should be with him during his last moment. We were so regretful and felt helpless. He passed away finally.*

(P25)

#### 3.2.2. Unhealthy Marital Relationship

Besides unfulfilled filial responsibilities, participants also mentioned that their marital relationships were affected during the pandemic. Eleven of the twenty-five participants described strained or deteriorating relationships primarily related to prolonged separation, financial losses, and inadequate communication during the pandemic. Besides this, their husbands’ underemployment or unemployment led to decreased monthly household incomes, which further induced family conflicts and indirectly damaged the harmony of the marriage. For example, one participant described a broken relationship with her husband.

*My husband had previously worked at a hotel but was terminated by his employer in March 2020. Since then, he could not find a full-time job. After that, we started to have more quarrels and conflicts than before due to financial problems. We are separated, and he moved out for a few months.*

(P13)

In addition to the financial problems, the prolonged separation due to the lockdown in Hong Kong may have limited couples’ intimacy, giving rise to an unhealthy marital relationship. A young housewife expressed her worries.

*My husband works in China on weekdays and comes back every Saturday. Apart from my daughter, I have no other relatives in Hong Kong. After having the lockdown policy, he stayed in China and could not come back for months. I’m so worried about our relationship as some of my friends divorced due to a long-time separation.*

(P6)

#### 3.2.3. Tension in the Parent–Child Relationship

Thirteen of the twenty-five participants fulfilled caregiving roles for their children during the pandemic, and eleven of them experienced conflict and tension in their parent–child relationships due primarily to children’s distance/online learning at home and the limited opportunities for outdoor activities.

One mother experienced stress and difficulties when supervising her child, causing an increase in the harshness of her parenting and greater tension in the parent–child relationship.

*After school closure several months ago, my 8-year-old daughter should have online learning at home. She was not interested and concentrated on her online learning. She is naughty and did not finish her home assignments as scheduled. I was so angry and punished her sometimes. I could not control myself, and I regretted beating her. Also, I’m not familiar with her school assignments and using a computer.*

(P5)

Another participant echoed this sentiment, and further described difficulties in resolving the tension:

*I’ve two naughty and energetic little boys. They are studying K2 and K3. They don’t understand why they should stay at home. They always request to go to the playgrounds or child centers.*

(P12)

### 3.3. Adaptation to Social Challenges

The third theme, “Adaptation to social challenges”, regards the external impacts on the female participants as citizens. The three subthemes identified were: avoidance of social activities due to the social distancing policy; changes in environmental and household hygiene practices; and underemployment/unemployment/resignation causing financial losses. The participants adapted to these social challenges to ensure their safety and safeguard their families.

#### 3.3.1. Avoidance of Social Activities Due to Social Distancing Policy

Due to the local government’s social distancing policy and the closure of public facilities, eighteen participants showed understanding and mainly stayed at home, avoiding social activities or gatherings. Although nearly all outdoor activities were suspended, the participants had their own means of maintaining necessary social interactions and family connections. One participant described her approach as follows:

*I like swimming, table tennis, shopping and traveling with my family or friends. But I have seldom gone out with them in recent months because of the closure of the public facilities and the social distancing policy. Although we had reduced social gatherings, we maintained contacts via phone or social media networks.*

(P7)

Another participant supported the social distancing policy, understanding its importance in preventing the transmission of the disease. However, she emphasized concerns about her mother and elderly people with medical or mental health issues.

*I agree and support that we should minimize social gatherings or group activities to limit the spread of the disease. But I should regularly visit my mother as she has early dementia. Even though I could not visit her every day, I gave her a phone call daily. As a daughter, I think we should closely observe and care for our parents and those elderly in need, do not just leave them alone unattended.*

(P4)

#### 3.3.2. Environmental and Household Hygiene Practices Changes

Sixteen participants cleaned their households more often, or changed their hygiene practices, during the pandemic to prevent the transmission of the disease. Twelve participants showed an awareness of the need to maintain environmental and household hygiene.

A mother with two children referred to using sanitizing products and wearing masks to prevent infection.

*You know, it’s not easy to buy sanitizing products for household cleansing and suitable masks for my kids to put on as there was inadequate supply in the early stage.*

(P17)

One housewife lived with her husband and was the caregiver of her parents, who were not living together. Although the additional cleaning was time-consuming and labor-intensive, these actions increased her sense of safety.

*I have comprehensive household cleansing increased to twice a day instead of twice a week before. After every shopping or visiting my parents, I take a shower, thoroughly cleanse my body and sanitize my clothes. When I go out, I try to avoid touching public facilities and decrease visits to the wet market.*

(P9)

#### 3.3.3. Underemployment/Unemployment/Resignation Causing Financial Losses

In this subtheme, we can see that the employment opportunities of the participants or their husbands largely affected their financial situation. Of the 25 participants, 12 had full-time (3) or part-time jobs (9) before the pandemic. Ten of them had their voluntary or involuntary work reduced or terminated. Seven were underemployed or unemployed. Three resigned from their job, as they perceived their responsibility for taking care of their children was greater. Two of these three participants divorced, and one was separated from her husband. Some participants also reported that the additional expenses of protective masks, sanitizing products, or online-learning resources for their children further increase their financial burden.

A retired participant who was divorced with one daughter described the challenges as below:

*Being the mother, I should care for my daughter at home during the pandemic. Thus, I resigned from my part-time job. Only the monthly allowance from the government supports our daily expenses!*

(P3)

Another participant (separated from her husband) complained that her monthly income was reduced, but her expenses had increased.

*My husband has been underemployed for a few months with decreased salary. However, my son has online learning at home. In these few months, we should have extra expenses to purchase protective masks, sanitizers, cleansing products, and my son’s online learning equipment.*

(P19)

## 4. Discussion

Our findings show that the impacts of the COVID-19 pandemic on Hong Kong women were substantial. Even during the pandemic, they perceived family caregiving as their highest obligation, given their role as daughter, wife, or mother (or combinations thereof). All the themes and subthemes identified are interconnected. No matter the participants’ roles in life, they encountered personal (psychological and physical), familial, societal, and cultural challenges during the COVID-19 pandemic.

### 4.1. Deterioration of Personal Health

Most participants (22 out of 25) experienced a physical or psychological health deterioration, or both. They articulated a fear of being infected, or of their family being infected, along with the other indirect consequences of COVID-19. As in the previous online survey, the respondents had high levels of fear and perceived strong influences related to COVID-19 [10]. Additionally, the participants in this study came to understand the direct or indirect negative consequences of the pandemic through social media and news information. A local survey of 500 citizens in 2020 found that most respondents were afraid of becoming infected, and suspected they were infected [14]. There was a negative association between fear of infection and health-related quality of life [14]. Another local study investigating the psychological burden felt by middle-aged women during the SARS outbreak reported that high emotional stress was significantly associated with feeling scared, disrupted sleep, and financial losses [8]. Most Hong Kong citizens adopted good hygiene habits after SARS, and began wearing face masks in public areas when sick [8]. However, the unexpected global need for face masks and personal protective items in early 2020 caused supply shortages, leading to an increase in the fear of Hong Kong citizens [12]. Citizens who could not access adequate stock of face masks or sanitizer may have felt a greater fear of infection. A recent systematic review and meta-analysis of 44 articles found that respondents’ fear of COVID-19 was higher in Asia [15]. The provision of clear information related to COVID-19, along with psychological support services, could ease women’s fear and stress.

The participants mentioned increases in caregiving responsibilities under lockdown conditions, which further aggravated their burdens. The study participants who were family caregivers felt stress and physical exhaustion due to a lack of sufficient rest when performing their caregiving roles non-stop during the months of the pandemic. Evidence has shown that informal family caregiving roles are often demanding, and negatively affect caregivers’ physical and emotional health [16,17,18]. Motivations and enthusiasm for informal caregiving are significantly based upon cultural and societal matters [18]. Caregiving is commonly perceived as women’s responsibility, especially in Chinese culture. Liu and Dupre found that Chinese women who provide one or more hours of care for their parents show poor health levels compared to non-caregivers [3]. Another recent study identified an adverse impact on the wellbeing of Chinese women who provided long-term care for elderly parents at home [19].

Nowadays, there is a greater prevalence of nuclear families, with a smaller domestic household size in Hong Kong than before. The local government’s statistics show that the average domestic household size was 2.7 at the time of the study [20]. Thus, the family caregivers, especially the women, may be responsible for intergenerational care. Given that schools and childcare and elderly care centers were closed in mid-2020 in Hong Kong, our study participants’ caregiving responsibilities further intensified, increasing the caregiver burden, which echoes the findings of Connor et al.’s study [7]. Even though some of our study participants were aware of their own physical exhaustion, they continued their caregiving roles and showed unconditional love for their families. Thus, caregiving’s impact on women’s health and quality of life should be addressed during public health crises. If family caregivers become sick, infected, or have to enter quarantine, those under their care may suffer. The careful consideration of local governments or healthcare professionals in providing adequate support for caregivers’ physical and mental wellbeing are recommended, such as the provision of respite services.

### 4.2. Unfavourable to Family Relationships

In some Asian countries, filial piety and parental care are important traditional concepts [19]. The virtues of filial piety are established and cultivated in people at an early age via school education, family teaching, etc. [18]. Our study found that Hong Kong women perceived and accepted their roles and responsibilities related to caring for their parents, regardless of whether they lived with them. Daughters play a crucial role in family caregiving, while sons and daughters-in-law are vital family caregivers of parents in Chinese culture [21]. Holroyd conducted ethnographic research, and interviewed 20 caregiving daughters in Hong Kong [22]. Her results show that Chinese daughters have a strong sense of their caregiving obligations towards frail elderly parents, which is consistent with our findings. Although a few participants in this study had committed their parents to elderly care settings, they loved their parents, and visited them regularly. In the past, placing parents in an elderly care attention home was regarded as non-filial. Nowadays, though, it is widely recognized as an adequate substitution for filial piety in urban areas [21], as in Hong Kong.

Similar to other countries, the Hong Kong Special Administrative Region’s government enforced social distancing measures and a lockdown policy during the pandemic [23]. Thus, this study’s participants expressed regret that they could not visit their parents who resided in elderly care institutions, or who lived in nearby cities in mainland China. Ho and his team reported that the COVID-19 pandemic caused loneliness in elderly people in care institutions [24]. Two study participants regretted not being present during their parents’ dying moments due to the lockdown. The presence of children during their parents’ final moments is an integral part of filial piety in Chinese tradition [25,26]. Providing emotional support and physical connection are deemed essential to the parents’ “good death” [25]. A recent review reported that filial piety is worthwhile to promote and support. It can reduce the stresses and burdens of adult Chinese offspring, thus improving their sense of spirit and caregiving satisfaction [27]. Similarly, Western women are generally their parents’ caregivers [28]. Although cultural expectations have tended to differ between Eastern and Western countries, the elements of filial piety have become more alike throughout global changes and evolution [27,29].

Besides unfulfilled filial responsibilities, unhealthy marital relationships represent another significant issue felt by families during the pandemic. Our study participants related their marital experiences and challenges, such as separation and financial losses due to the pandemic and lockdown. The experience of broken or deteriorated relationships is consistent with the findings of Maiti et al. [30]. They identified several issues that may interfere with married couples’ harmonic relationships during a pandemic, such as inappropriate communication, overworking, unemployment or salary reduction, relationship breakdown or even later divorce, etc. [30]. Indeed, the findings are not only specific to Chinese women, but could apply across countries and cultures. Prime et al. stated that significant life events could intensify pre-existent marital dissatisfaction, resulting in relationship breakdowns or even divorce [31]. A recent study review conducted in various countries, including Singapore, Canada, Spain, and Australia, found that reduced marital intimacy could further intensify the probability of domestic violence [32]. In contrast, some of our participants experienced improved relationships, showing how good communication with the sharing of emotional needs and bonding is required during times of hardship [32,33].

Due to the closure of schools and study centers in 2020 in Hong Kong, parents, especially mothers, had to take up some responsibilities related to their children’s learning from school teachers. The study participants experienced stress and difficulties in facilitating their children’s distance or online learning at home. They found that their children had problems finishing their assignments on time, mainly due to insufficient study interest and concentration. Sonnenscheina and Stites reported that parents’ stress levels and technology competency largely affect children’s involvement in online/distance learning [34]. A local online study assessed 6702 parents, 93% of whom were mothers, regarding their views on young school children’s distance learning [35]. Inadequate support and home–school communication were the parents’ foremost complaints. Besides this, the impact of the pandemic and stay-home policies increased parental stress, damaging the parent–child relationship and increasing the possibility of harsh parenting [36], which echoes our findings. Thus, it was recommended the schools offer adequate technological support, strengthen communication with parents, and offer flexible learning activities. Several study participants (mothers of young children) stated that they occasionally lost their temper. They regretted inappropriate actions taken towards their children, which agrees with the study findings of Sonnenscheina and Stites [34], revealing that parents might experience unstable moods and conflicting feelings under lockdown. The authors also suggested that parents could stabilize their emotions by performing other activities with their children, and thus avoiding conflict. Campbell [37] also highlighted that child abuse or neglect might be underreported during the pandemic.

### 4.3. Adaptation to Social Challenges

Apart from individual and relational impacts, the participants also encountered external impacts, demonstrating a certain degree of adaptation to social challenges. The social distancing policies of the Hong Kong Special Administrative Region Government aimed to reduce the possibility of COVID-19 spreading in the community [38]. The majority of study participants agreed with and supported minimizing social activities and performing personal and environmental hygiene practices. Indeed, most Hong Kong citizens were already aware of their infection control responsibilities, and were willing to take preventive measures and undertake social distancing and environmental hygiene practices [39,40]. Evidence has shown a positive correlation between fear of COVID-19 and changes in public health behavior, as greater fear enhances citizens’ compliance with public health behaviors, such as maintaining social distancing, mask-wearing, and hygiene practices [10,41,42].

Due to the closures of schools, childcare centers, and playgrounds, most women caregivers showed concern over whether their children would have adequate support and care at home [2,43]. Women caregivers may reduce their working hours and withdraw from the labor market [44]. Among the twelve participants who had full-time or part-time jobs before the pandemic, seven had voluntarily or involuntarily reduced their working hours, and three had resigned from their jobs, as they perceived their caregiving roles at home to be of greater significance. These ten participants’ family income and financial status were significantly affected. According to the Census and Statistics Department of Hong Kong [45], the number of unemployed females significantly increased to 92,400 (5.7%) in 2020, which is more than double the figure before the COVID-19 pandemic in 2019 [45]. The number of those underemployed increased to 54,700 in 2020, which is about five times greater than the 11,300 in 2019 [45]. Besides this, the additional expenses required for protective masks, sanitizing products, or online-learning resources, and reductions in families’ monthly household incomes, could cause significant financial burdens. Although the local government has implemented a series of anti-virus policies, including establishing an Anti-Pandemic Fund, to meet the challenges facing society [23], continuous and comprehensive support should not be neglected.

Women’s self-perceptions and actions are essentially formed out of social and cultural circumstances [46]. Although Hong Kong is now a modern urbanized city, our study has demonstrated that Hong Kong women still generally preserve traditional cultural values and duties, echoing the findings of Han [46]. The traditional Chinese view of the significance of family is evident through the female Hong Kong participants’ behaviors. These behaviors show that the Chinese women adhered to familial duties and responsibilities in order to maintain family concord and stability [46]. Based on the tradition of filial piety, there was a feeling of mutual responsibility among parents and children. Children cared for their parents. In turn, parents are devoted to loving their children [46], which explains our participants’ displays of selfless sacrifice and unconditional love for their families during the pandemic.

The COVID-19 pandemic gave rise to various physical and psychological health impacts and general life challenges for Hong Kong Chinese women. To support the women who have been substantially affected by the pandemic, local governments, non-governmental organizations, and healthcare professionals should consider strategies and activities that will enhance women’s physical and mental well-being, and help them tackle the life challenges experienced during hardship. For instance, online counseling services or telephone hotlines for psychological support, and instant preventive and protective measures regarding COVID-19, may help reduce their fear. It is also recommended to increase the anti-pandemic financial assistance available for those who have lost their jobs or are underemployed, as well as to provide domestic help, childcare, or elderly respite services for those in need.

### 4.4. Limitations

This study has several limitations. Firstly, the selection of participants. Participants were recruited voluntarily from a pool of 417 Chinese women who had previously responded to an online survey, and who were primarily members of Hong Kong Federation Women Centres. Another limitation is the small sample size, comprising 25 participants from different centers, which may not be sufficiently representative for drawing generalizable conclusions. However, the objective of the interpretive phenomenological approach is to describe, understand and interpret participants’ experiences [11]. Carefully identifying the subjects with wide variations and information-rich can facilitate a more in-depth understanding [47].

## 5. Conclusions

The COVID-19 pandemic has had unpredictable and prolonged impacts on human health, society, the economy, and the environment worldwide. Understanding the health impacts and life challenges related to the pandemic for Hong Kong women will help improve their resilience and mitigate adverse consequences. This study’s findings illustrate that Hong Kong women perceived family caregiving as their paramount obligation during the pandemic. The pandemic increased their awareness of their caregiving roles, and has diminished their quality of life. Apart from re-thinking the sharing of care duties among family members in order to relieve women’s burdens, the promotion of strategies or activities that could enhance women’s physical, psychological, emotional and social health, and general quality of life, is recommended. Comprehensive and continuous support from the local government, non-governmental organizations, and healthcare organizations is required.

## Figures and Tables

**Table 1:** Demographic information of participants (*n* = 25).

No.	Age Group	Marital Status	Working Status before (during) COVID-19	Caregiving Role (Caregiver of)
1	60–69	Single	Retired	Yes (father)
2	60–69	Widowed	PT*	No
3	30–39	Divorced	PT (resigned)	Yes (daughter)
4	50–59	Married	PT	Yes (mother)
5	40–49	Separated	PT (resigned)	Yes (daughter)
6	30–39	Married	Housewife	Yes (daughter)
7	40–49	Divorced	PT (resigned)	Yes (mother and daughter)
8	60–69	Married	Retired	Yes (husband and daughter)
9	40–49	Married	Housewife	Yes (father and mother)
10	30–39	Married	FT^#^ (underemployment)	Yes (daughter)
11	30–39	Married	PT (unemployment)	Yes (mother-in-law and son)
12	30–39	Married	PT (underemployment)	Yes (two sons)
13	30–39	Separated	PT (underemployment)	Yes (son)
14	60–69	Single	Retired	Yes (niece)
15	60–69	Married	Retired	Yes (husband)
16	30–39	Married	Housewife	Yes (son and daughter)
17	30–39	Separated	FT (underemployment)	Yes (two daughters)
18	20–29	Married	Housewife	Yes (son)
19	30–39	Separated	Housewife	Yes (son)
20	60–69	Married	PT (underemployment)	No
21	50–59	Married	Housewife	No
22	30–39	Single	FT (unemployment)	No
23	50–59	Married	Housewife	Yes (husband)
24	60–69	Married	Retired	Yes (husband)
25	60–69	Married	Housewife	Yes (father and mother)

FT^#^—full time, PT*—part-time.

**Table 2:** The main theme, the three interconnected themes, and the eight subthemes.

Core Theme	Interconnected Themes	Subthemes
Perceived family caregiving as paramount self-obligation in times of COVID-19 pandemic	Deterioration of personal health	Emotional and psychological distress due to fear of COVID-19′s consequences
		Physical exhaustion in fulfilling the family caregiver role during COVID-19
	Unfavorable to family relationships	Regrets of unfulfilled filial responsibilities
		Unhealthy marital relationship
		Tension in the parent–child relationship
	Adaptation to social challenges	Avoidance of social activities due to social distancing policy
		Environmental and household hygiene practice changes
		Underemployment/unemployment/resignation causing financial losses

## Data Availability

The data presented in this study are available on reasonable request from the corresponding author.
